# IROC phantoms accurately detect MLC delivery errors

**DOI:** 10.1002/acm2.70017

**Published:** 2025-02-18

**Authors:** Sharbacha S. Edward, Julianne M. Pollard‐Larkin, Peter A. Balter, Rebecca M. Howell, Christine B. Peterson, Stephen F. Kry

**Affiliations:** ^1^ Department of Radiation Physics The University of Texas MD Anderson Cancer Center Houston Texas USA; ^2^ Department of Biostatistics The University of Texas MD Anderson Cancer Center Houston Texas USA; ^3^ Department of Radiation Physics Outreach The University of Texas MD Anderson Cancer Center Houston Texas USA

**Keywords:** dosimetry, MLC, QA

## Abstract

**Purpose:**

We evaluated the impact of random and whole‐bank multileaf collimator (MLC) delivery errors on dosimetric delivery accuracy in the Imaging and Radiation Oncology Core (IROC) phantom audits, as well as differences in delivery accuracy between the IROC phantom prescription and typical clinical fraction sizes.

**Methods and Materials:**

Plans were created for the IROC IMRT head and neck (H&N) and SBRT spine phantoms. MLC leaf errors were introduced into the plans: random shifts between −2 and 2 mm, and whole bank shifts of 0.5, 1, and 2 mm. Plans were recalculated and delivered on a Varian Truebeam, and the log files were analyzed using Mobius Fx software. A second study examined the impact of fraction size on MLC position accuracy and corresponding dose delivery accuracy. The standard IROC phantom prescriptions (∼6 Gy) were scaled to the extremes of 2 Gy for H&N and 27 Gy for spine. All plans (original and scaled) were delivered on a Varian Truebeam and 21EX machine.

**Results:**

Random MLC positioning errors produced small average dose deviations in the PTV of up to −2.8% for H&N and 0.7% for spine. Whole‐bank MLC shifts resulted in larger average PTV dose deviations up to 8% for H&N and 7.1% for spine. The Varian 21EX irradiations had greater MLC root mean square (RMS) error than Truebeam plans. Plans with smaller prescriptions (and faster leaf motion) had greater MLC RMS errors, but plan accuracy was not affected dosimetrically – all results remained within 1% regardless of fraction size.

**Conclusions:**

Both random and whole bank MLC shifts caused dose deviations in the IROC phantoms that were comparable to clinical results previously found in the literature. Deviations measured with ion chambers were well matched with delivery log file analysis. Smaller dose‐per‐fraction prescriptions caused larger MLC RMS errors that were detected with log files, but were clinically insignificant compared to the dosimetric accuracy of the plan.

## 1 INTRODUCTION

The Imaging and Radiation Oncology Core (IROC) phantom program has been used internationally as an end‐to‐end test of the patient radiation treatment process for decades. Several phantoms comprise this program, including the intensity‐modulated radiation therapy (IMRT) head and neck (H&N) and stereotactic body radiation therapy (SBRT) spine phantoms. Treatment delivery accuracy is assessed using planned versus measured dose readings, and continues to show a need for improvement across the field: the IMRT H&N phantom has a current pass rate of ∼92%^1^ and the SBRT spine phantom has an average pass rate of ∼83%.^2^

In previous work, we showed that dose calculation errors played a major role in phantom failures and dose deviations seen among H&N and spine phantom irradiations.^3^, ^4^ In particular, the multileaf collimator (MLC) modeling parameters (DLG or MLC‐offset) were critical to accurate dose delivery.^5^ In this work we examined how MLC positioning (MLC_P_) errors, that is, a difference between intended and actual MLC position, impact the phantom result. In a multi‐institutional study, MLC_P_ errors averaged 0.46 mm for VMAT treatments, 0.32 mm for dynamic (DMLC) treatments, and 0.008 mm for step‐and‐shoot treatments.^6^ Systematic MLC_P_ errors, such as offsets in the MLC leaf bank have been shown to cause changes to the planning target volume (PTV) dose of 3.7% and 7.2% for offsets of 1 and 2 mm respectively.^7^ Random leaf error effects have been found to be smaller, causing dose errors of up to 1.6%.^8^ While these delivery errors have been examined in patient cases, it is unclear how such effects would manifest in a standardized credentialing phantom. It is important to understand how MLC_P_ errors would manifest as delivery errors in IROC's phantoms due to the ongoing high rate of phantom failure in the community and the possibility of MLC_P_ errors being a relevant failure mode.

Additionally, IROC phantoms are all irradiated to a single fraction dose of approximately 6 Gy.^1^, ^3^ This is to optimize the dosimetry, but this fraction size is different from clinical fraction doses which are typically 2 Gy for IMRT H&N and range from 8 Gy to well in excess of 20 Gy for SBRT spine treatments.^9^, ^10^, ^11^, ^12^ During the delivery of dMLC and VMAT treatments, the MLC is constantly moving, and this motion is influenced by the length of the arc, dose rate and number of MUs to be delivered. When the dose per fraction is decreased (for the same treatment plan) the mean MLC speed increases because of the motion required throughout the delivery. MLC leaves move more quickly during a low‐dose delivery because there is a shorter time frame within which the full plan modulation must occur. This quicker MLC motion results in a greater possibility of MLC positional error (a greater RMS error), which may result in treatment delivery discrepancies. If these effects are substantial enough, the IROC phantom (using a 6 Gy prescription) may miss an actual failure (using a 2 Gy prescription) because the leaves are moving artificially slowly for the phantom case. The opposite is also true, slower MLC motion may result in less MLC error and the IROC phantom (using a 6 Gy prescription instead of a much larger prescription) may be inappropriately sensitive compared to a clinical plan that will have less MLC error.

In this work, we evaluated the impact of random, whole bank and fraction size MLC delivery errors on IROC phantom irradiations.

## 2 MATERIALS AND METHODS

### 2.1 Phantoms and plans

**FIGURE 1:**
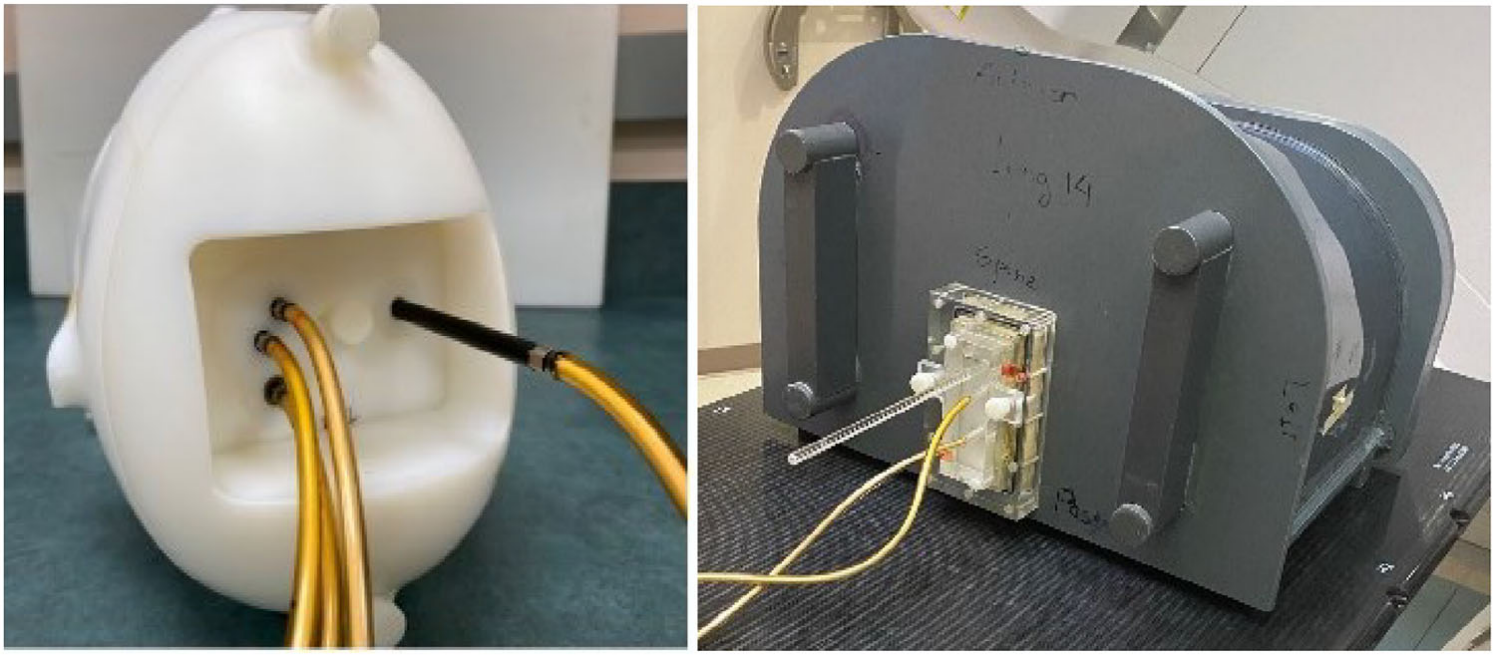
IROC phantoms modified to hold ion chambers (A1SL scanning chambers) at the traditional location of the TLD. IMRT head and neck (left). SBRT spine (right). IROC, Imaging and Radiation Oncology Core.

The 2 phantoms used for this study were the IROC IMRT head & neck (H&N) and SBRT spine anthropomorphic phantoms. Both phantoms are composed of tissue‐equivalent materials and are used as an end‐to‐end test of patient radiation treatment. To improve dosimetric precision, the phantoms were modified so that the inserts held Exradin A1SL ion chambers in the target and OAR structures instead of the usual TLDs (Figure 1).

The H&N phantom consists of 2 PTVs, both 5 cm long. The primary PTV representing a tumor, is 4 cm in diameter and holds 2 ion chamber (anterior and posterior). The secondary target representing a lymph node, is 2 cm in diameter and holds 1 ion chamber. One chamber is also inserted into the OAR (cord).^1^, ^13^ The spine phantom simulates the human thorax, with a 22 cm^3^ target sitting on the vertebral body, approximately 0.8 cm from the spinal cord. The spine insert accommodates 1 ion chamber in the PTV and 1 in the OAR. (Figure 1).

All plans were created by the same individual, using the collapsed cone algorithm on the Raystation 10B (Raysearch) treatment planning system. Both segmented multileaf collimator (sMLC), also known as “step and shoot” and VMAT plans were created for each phantom. These modalities were selected to span the range of observed MLC_P_ errors in clinical delivery, from least error with step and shoot to largest error with VMAT.^4^ Plans were developed following the IROC irradiation dose prescription of 6.6 Gy for the H&N and 6 Gy for the spine. All plans were developed at 6 MV, the sMLC plans each had 9 beams and the VMAT plans had 2 arcs. Plans were all created and optimized to spare organs at risk while meeting the prescription dose coverage to 95% of the PTV. For all plans, the maximum leaf speed was set to 2.25 cm/s, the minimum segment size was 4 cm^2^, the minimum dynamic tip gap was 1 mm, and the dose grid resolution was 3 mm.

### 2.2 Introduced delivery errors

MLC_P_ errors were introduced into the phantom plans to explore the impact of delivery errors on the IROC phantom irradiation process. Various combinations of random leaf and whole leaf bank MLC shifts were introduced to each plan using the scripting function in Raystation. A random number generator was used to select random leaves to shift on either leaf bank. The leaf shift distance was then randomly picked from the following: −2, −1, −0.5, 0.5, 1, or 2 mm. Different perturbed plans were created with shifts introduced to either 25%, 50%, 75%, or 100% of leaves. To focus on MLCs errors that could have a dosimetric impact on the plan and would be measured with the ion chambers, these shifts were implemented only among leaves that span the PTV. For the sMLC plans, shifts were assigned randomly to leaves in each segment of each beam. Shifts in the VMAT plans were assigned to a random leaf in the first segment and that shift was maintained through all segments of the beam, to simulate a lagging leaf scenario. Whole bank leaf shifts were introduced as either 0.5, 1‐ or 2‐mm total shift outwards (opening of banks), with each bank moving half the distance. All plan doses were recalculated with the new “erroneous” MLC positions, resulting in 32 total plans). Average dose values for the PTV and cord ion chambers were recorded from Raystation for both the original and altered plans.

### 2.3 Different prescription sizes

Additional VMAT plans were created with single fraction sizes of 2 Gy for H&N and 27 Gy for spine, to test lower and upper limits of dose per fraction effects on the phantom delivery. To maintain consistency in plan complexity, the plan doses were scaled to the IROC prescription and complexity metrics including modulation complexity score,^14^ plan modulation and plan irregularity,^15^ were checked to ensure that they remained the same, indicating consistency between scaled and unscaled plans. This resulted in 8 total plans (2 for each machine: TrueBeam and 21EX, for each phantom).

### 2.4 Delivery and analysis

The two machines used were the Varian Truebeam (Varian Medical Systems, Palo Alto, CA) with a high‐definition MLC (HDMLC) and the Varian 21EX linear accelerator with Millennium 120 MLCs (Table 1). Both machines were evaluated because the Truebeam consists of better integrated digital technology than the C‐series machines, including the Maestro electronic controller system.^16^ Both machines were located at the MD Anderson clinic in Houston, Texas, where they are used for patient treatments and regularly checked and serviced as part of routine clinical operations and maintenance. The plans for the first part of this study which examines random versus whole bank MLC_P_ errors were irradiated on the Truebeam machine, while the plans with varying prescription sizes were irradiated on both machines, to identify differences between the two generations of technology. All plans were delivered with a dose rate of 600 MU/min.

**TABLE 1:** MLC characteristics for varian truebeam and 21EX machines.

Machine	Inner MLC pairs	Outer MLC pairs
Varian Truebeam (HDMLC)	0.25 cm (*n* = 32)	0.5 cm (*n* = 28)
Varian 21EX	0.5 cm (*n* = 40)	1.0 cm (*n* = 20)

Delivery log files were collected for each plan and processed, which provides details of dose delivered and RMS errors detected in the MLCs. The impact of both random and systematic (whole bank) leaf errors on the phantom plans were examined by comparing treatment planning system (TPS)‐original to TPS‐perturbed plan doses. The treatment log files were processed using the Mobius Fx software (V2.1.2), to confirm TPS predicted dose deviations. The dosimetric impact of fraction size was examined by comparing measured versus TPS doses and the log files were analyzed to assess MLC RMS error differences between the pairs of plans (original vs. scaled).

## 3 RESULTS

### 3.1 Impact of introduced leaf errors on phantom dose

**FIGURE 2:**
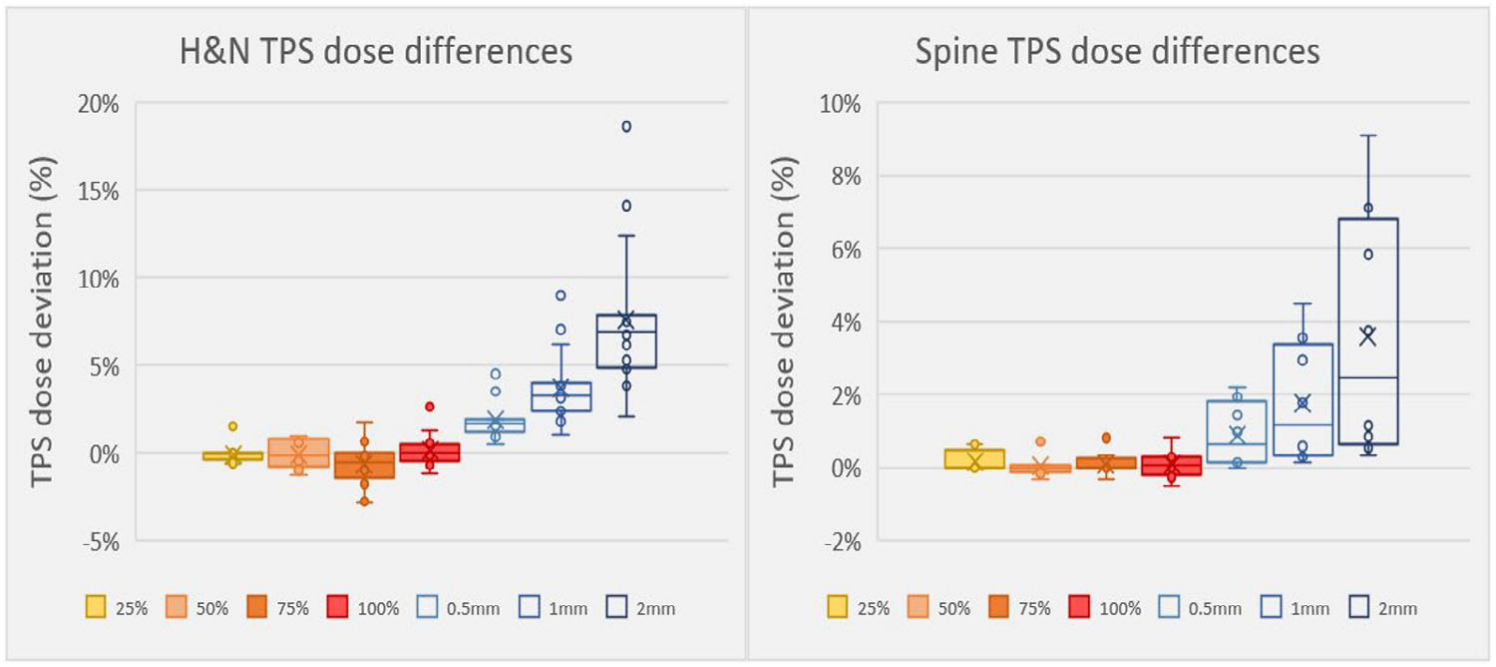
Dose deviations of phantom plans due to random and systematic MLC error. The solid boxes represent the random shifts, where the % marker denotes the percent of leaves shifted. The hollow bars represent the systematic (whole bank) MLC shifts. MLC, multileaf collimator.

TPS dose comparisons between plans showed dose deviations for both random and systematic MLC errors. Random MLC shifts resulted in relatively modest dose deviations, of up to −2.8% in the PTV (cord: also −2.8%) for the H&N phantom and 0.7% (cord: 0.8%) for the spine. Systematic shifts of the entire leaf bank resulted in much larger deviations. As the leaf bank offset increased, the phantom showed increasing dose deviations, with 2 mm shifts leading to PTV dose differences in the H&N phantom of up to 8% (cord: 8.7%) and 7.1% (cord: 9.1%) in the spine (Figure 2). Random versus whole bank shift mean dose deviations were statistically significant for both phantoms: H&N (*p* < 0.001) and spine (*p* = 0.009), reinforcing the importance of systematic bank offsets.

**FIGURE 3:**
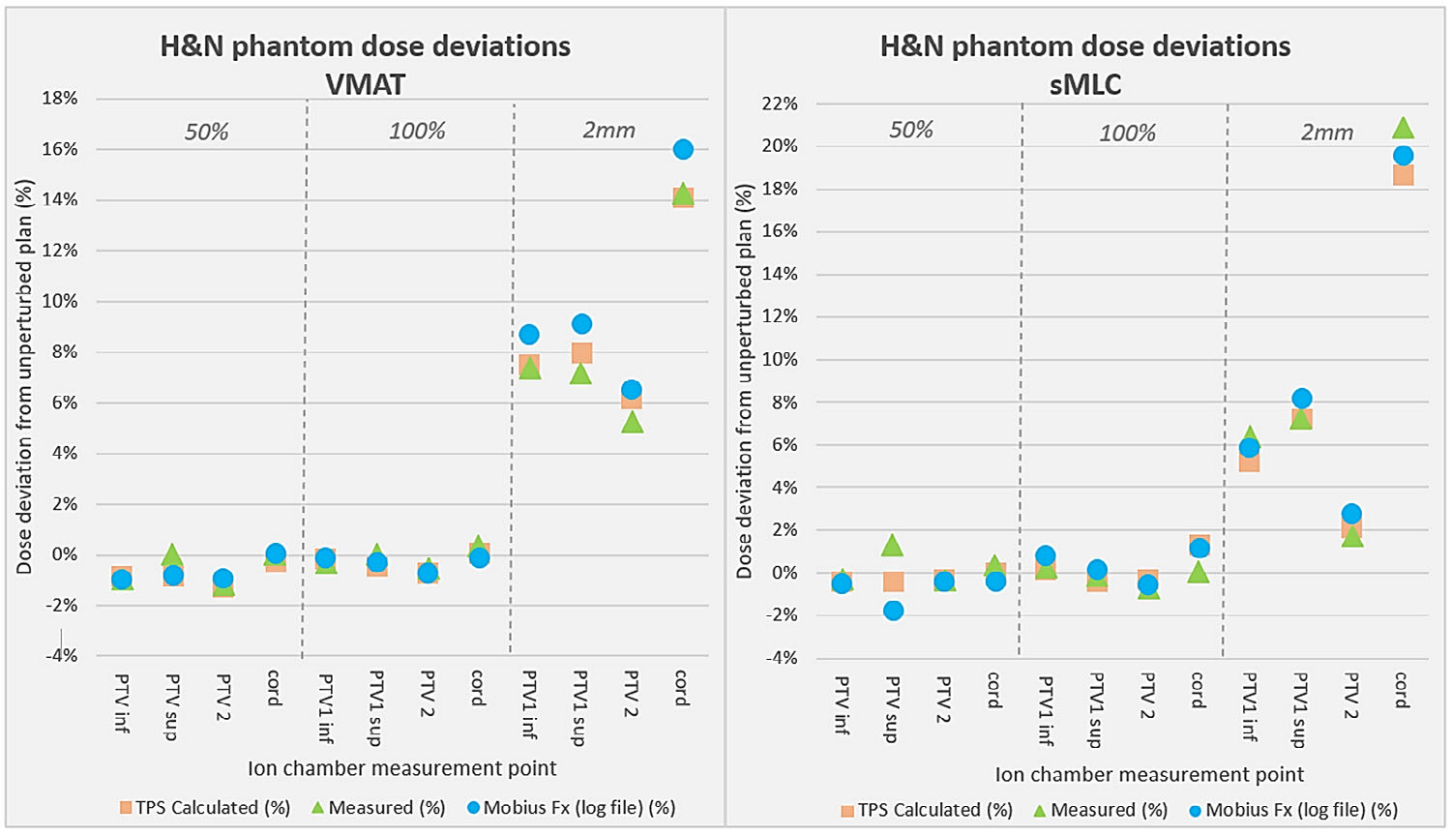
Dose comparisons between TPS, measured (ion chamber), and delivered (log file) doses for the H&N phantom, showing differences in recorded dose deviation across all 3 modes. Dose deviations represent differences from the original unperturbed plan, for the 3 plans that were irradiated: 50%, 100% and 2 mm MLC shifts. Each datapoint represents an ion chamber point on the phantom; 3 plans each for VMAT and sMLC, with 4 ICs each. MLC, multileaf collimator; sMLC, segmented multileaf collimator.

**FIGURE 4:**
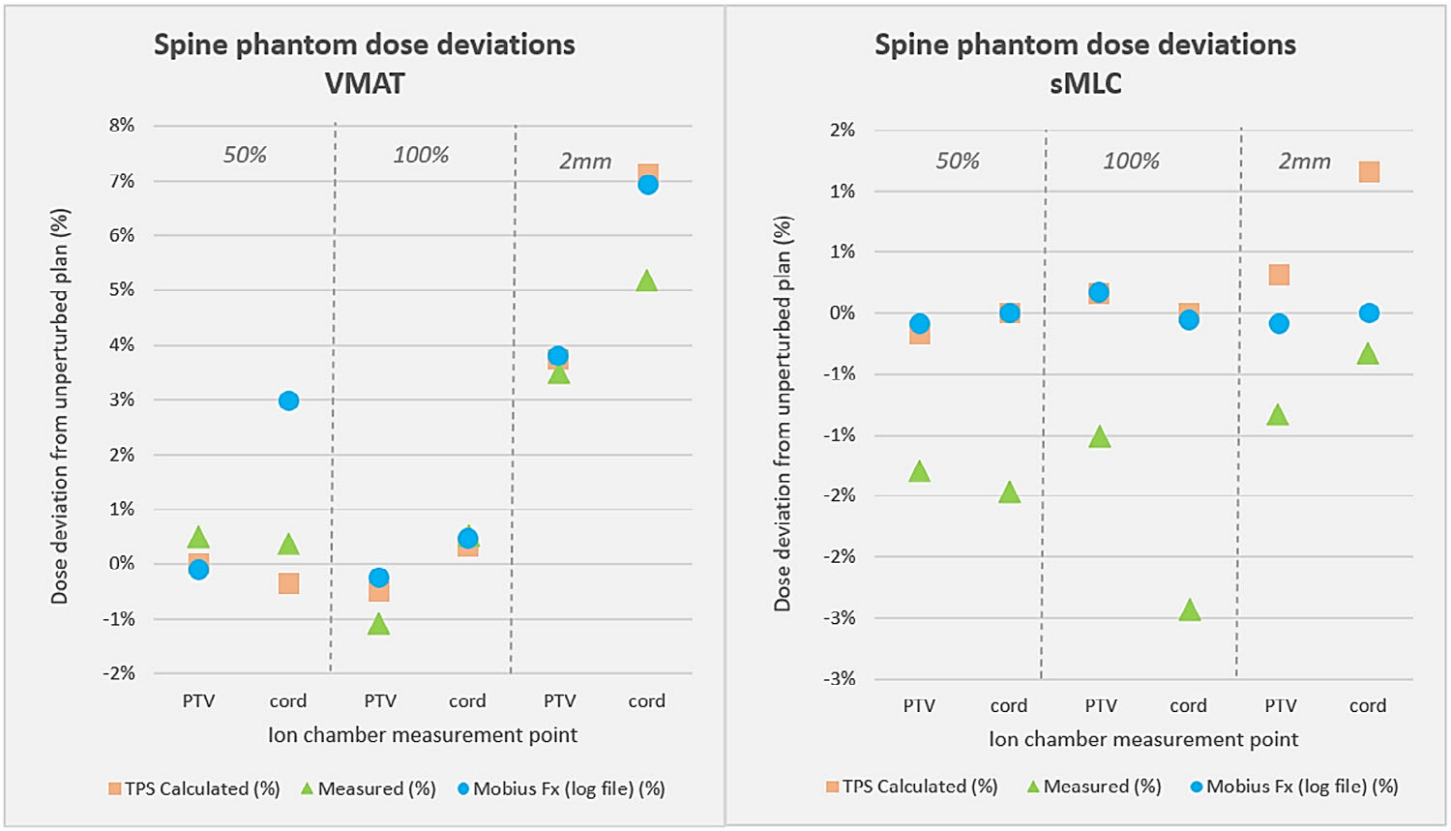
Dose comparisons between TPS, measured (ion chamber), and delivered (log file) doses for the spine phantom, showing differences in recorded dose deviation across all 3 modes. Dose deviations represent differences from the original unperturbed plan, for the 3 plans that were irradiated: 50% and 100% of leaves randomly shifted and 2 mm systematic leaf shifts. Each datapoint represents an ion chamber point on the phantom; 3 plans each for VMAT and sMLC, with 2 ICs each. Note the reduced dose scale (*y*‐axis) of the sMLC data. sMLC, segmented multileaf collimator.

TPS‐predicted dose deviations between the plan pairs (TPS‐original to TPS‐perturbed) were compared to the measured (Ion chamber: IC) and delivery log file (MFx) dose deviations for both phantoms. Comparisons were made for each ion chamber location, for each phantom, to get more detailed results across the phantom geometry. For the H&N phantom, dose deviations predicted by the TPS, measured by the IC, and calculated by MFx were within 2% of each other in 21 of 24 sampled points across the PTVs and OAR (Figure 3). For the spine, dose deviations were within 2% of each other in 10 of 12 sampled points (PTV & OAR), and at all points within the PTV (Figure 4). Overall, the average disagreement between the TPS and measured dose perturbation was 0.72%, while the average disagreement between the delivery log calculation and measured dose perturbation was 0.89%. The agreement between the TPS and measured perturbations indicate that introduced errors were reasonably well modeled in the planning system, and also emphasize that randomly occurring RMS errors (as opposed to the ones intentionally introduced in this study) did not manifest as a notable delivery error for any of these plans. The agreement between these perturbations and the delivery log file predictions emphasizes that these errors were accurately captured with the delivery log system.

### 3.2 Fraction size

**FIGURE 5:**
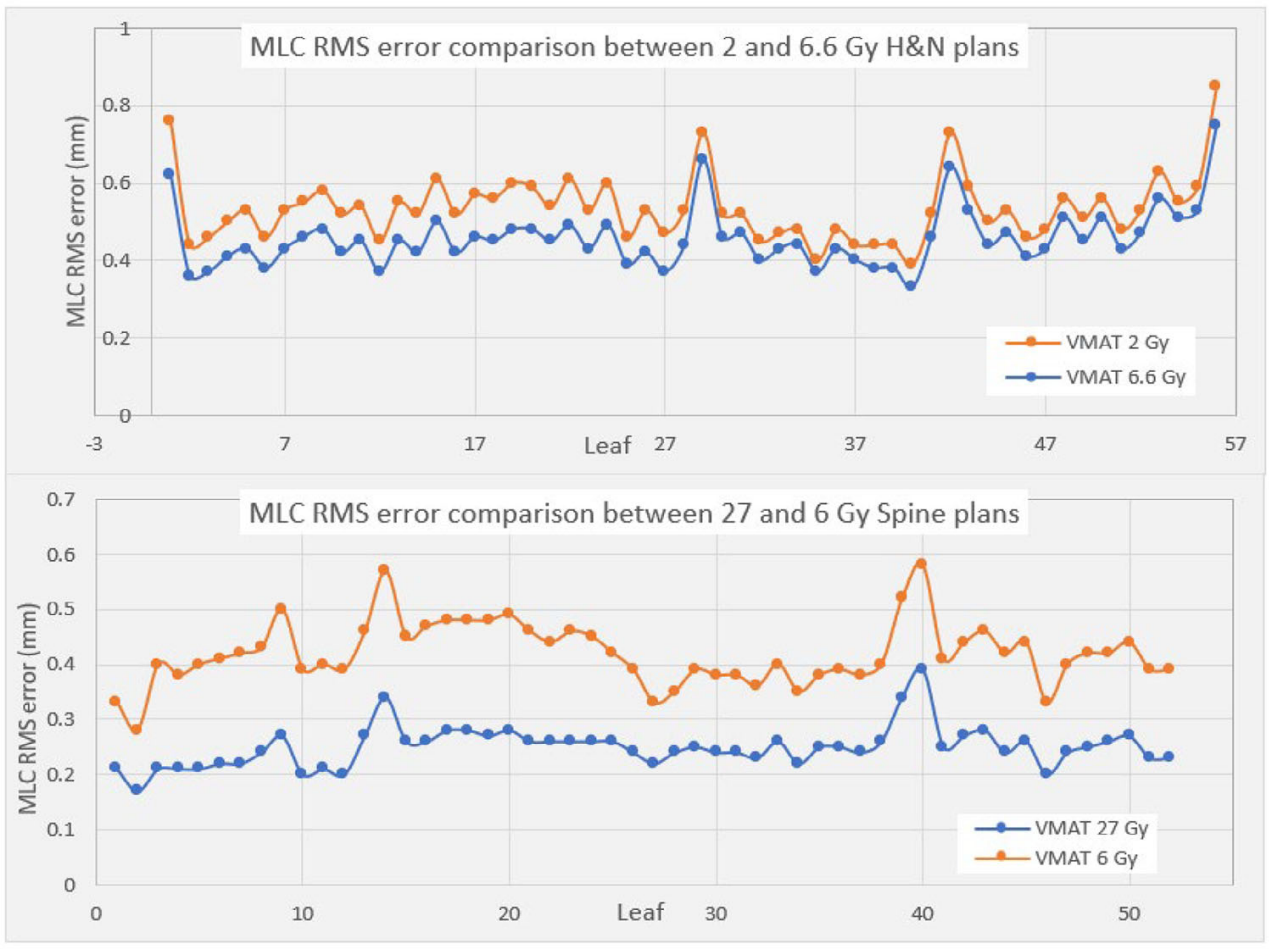
Distribution of all MLC RMS errors for the two VMAT plan pairs for the H&N (top) and spine (bottom) phantoms, irradiated on the Varian 21EX machine. H&N, head and neck; MLC, multileaf collimator; RMS, root mean square.

For the Truebeam plans, there was very little MLC RMS error found in any plan, regardless of the fraction size. All RMS error was within 0.05 mm for both dose‐per‐fractions in both the H&N and spine phantoms. The plans delivered on the 21EX machine, however, showed differences in RMS error between plan pairs. The H&N 2 Gy plan had 17% more RMS error on average than the 6.6 Gy plan (*p* < 0.001) and the spine showed an even greater difference, with the 6 Gy plan having 68% more error than the 27 Gy (*p* < 0.001). Leaf RMS error showed the same pattern of distribution throughout in each plan pair, with a distinct increase in the magnitude of error in the plans with a smaller prescription (Figure 5).

While the fraction size clearly impacted the MLC positioning accuracy in terms of random RMS error, the dosimetric impacts of these errors were negligible in all cases. When assessing the dosimetric accuracy of plan delivery, by comparing the delivered doses (from the log files) to the TPS dose, the maximum difference in accuracy was 1% for the 2 H&N plans, and 0.1% for the 2 spine plans (Table 2).

**TABLE 2:** Comparisons of plan delivery accuracy between the two plan prescriptions, showing similar plan delivery accuracy despite varying prescription size.

	Delivery dose deviation from TPS		
Head and neck	6.6 Gy	2 Gy	Difference in accuracy
PRIMARY PTV (1)	−0.09 (0%)	−0.02 (0%)	0%
PRIMARY PTV (2)	−0.07 (−0.1%)	−0.03 (0%)	0.1%
SECONDARY PTV	−0.04 (0%)	−0.01 (0%)	0%
CORD	0.03 (0.7%)	0 (0.9%)	0.2%

Spine	27 Gy	6 Gy	
PTV	−0.36 (−1.3%)	−0.08 (−1.3%)	0%
CORD	0.14 (0.9%)	0.03 (0.8%)	0.1%

## 4 DISCUSSION

The IROC H&N and spine phantoms were used to test the IROC H&N and spine phantoms’ ability to capture MLC_P_ errors during treatment delivery. The delivery log file analysis in Mobius Fx recorded dose deviations similar to the measured and TPS doses and can be reliably utilized as a secondary form of analysis for the IROC phantoms. Limitations do exist, however, where log files may not detect errors caused by poor leaf calibration, for example.^17^ This highlights the need for proper calibration of machine components such as MLCs and independent end‐to‐end testing to verify accuracy.

Analysis of VMAT plans with varying prescription doses demonstrated increased MLC RMS errors for the plans with smaller prescriptions. This was due to the MLC leaves having to move more quickly because of lower MUs and shorter delivery time for the gantry to circumnavigate the entire arc. Speeds were found to be up to 4 times faster for the spine 6 Gy versus the 27 Gy plan, and that resulted in 68% more RMS error. In our case, these RMS errors did not create a clinically impactful dose discrepancy. Given that these plans were irradiated on well‐maintained machines, these results represent the best‐case scenario for errors. Dose deviations may be greater on older machines with a less rigorous QA or maintenance schedule.

## 5 CONCLUSION

Dose deviations observed in the phantom were consistent with reported deviations observed in clinical cases, reinforcing that this phantom is a suitable patient surrogate and appropriate for clinical trial credentialing and QA.

## AUTHOR CONTRIBUTIONS

The authors confirm contribution to the paper as follows: study conception and design: Kry, Edward, Balter; data collection: Edward, Pollard‐Larkin; analysis and interpretation of results: Edward, Kry, Howell, Balter, Peterson, Pollard‐Larkin; draft manuscript preparation: Edward, Peterson, Kry. All authors reviewed the results and approved the final version of the manuscript.

## CONFLICT OF INTEREST STATEMENT

The authors declare no conflicts of interest.
